# Safety Assessment of Ultrasound-Assisted Intravesical Chemotherapy in Normal Dogs: A Pilot Study

**DOI:** 10.3389/fphar.2022.837754

**Published:** 2022-03-18

**Authors:** Noboru Sasaki, Yoshinori Ikenaka, Keisuke Aoshima, Teiichiro Aoyagi, Nobuki Kudo, Kensuke Nakamura, Mitsuyoshi Takiguchi

**Affiliations:** ^1^ Laboratory of Veterinary Internal Medicine, Faculty of Veterinary Medicine, Hokkaido University, Sapporo, Japan; ^2^ Translational Research Unit, Veterinary Teaching Hospital, Faculty of Veterinary Medicine, Hokkaido University, Sapporo, Japan; ^3^ Water Research Group, Unit for Environmental Sciences and Management, North-West University, Potchefstroom, South Africa; ^4^ Laboratory of Comparative Pathology, Faculty of Veterinary Medicine, Hokkaido University, Sapporo, Japan; ^5^ Department of Urology, Tokyo Medical University Ibaraki Medical Center, Ami, Japan; ^6^ Division of Bioengineering and Bioinformatics, Faculty of Information and Technology, Hokkaido University, Sapporo, Japan

**Keywords:** drug delivery, intravesical chemotherapy, microbubble, non-muscle invasive bladder cancer, pirarubicin, ultrasound

## Abstract

Intravesical chemotherapy after transurethral resection is a treatment option in patients with non-muscle invasive bladder cancer. The efficacy of intravesical chemotherapy is determined by the cellular uptake of intravesical drugs. Therefore, drug delivery technologies in the urinary bladder are promising tools for enhancing the efficacy of intravesical chemotherapy. Ultrasound-triggered microbubble cavitation may enhance the permeability of the urothelium, and thus may have potential as a drug delivery technology in the urinary bladder. Meanwhile, the enhanced permeability may increase systemic absorption of intravesical drugs, which may increase the adverse effects of the drug. The aim of this preliminary safety study was to assess the systemic absorption of an intravesical drug that was delivered by ultrasound-triggered microbubble cavitation in the urinary bladder of normal dogs. Pirarubicin, a derivative of doxorubicin, and an ultrasound contrast agent (Sonazoid) microbubbles were administered in the urinary bladder. Ultrasound (transmitting frequency 5 MHz; pulse duration 0.44 μsec; pulse repetition frequency 7.7 kHz; peak negative pressure −1.2 MPa) was exposed to the bladder using a diagnostic ultrasound probe (PLT-704SBT). The combination of ultrasound and microbubbles did not increase the plasma concentration of intravesical pirarubicin. In addition, hematoxylin and eosin staining showed that the combination of ultrasound and microbubble did not cause observable damages to the urothelium. Tissue pirarubicin concentration in the sonicated region was higher than that of the non-sonicated region in two of three dogs. The results of this pilot study demonstrate the safety of the combination of intravesical pirarubicin and ultrasound-triggered microbubble cavitation, that is, ultrasound-assisted intravesical chemotherapy.

## Introduction

Bladder cancer is the second most common urological cancer, and more than 70% of patients have non-muscle invasive bladder cancer (NMIBC) at the initial diagnosis (Antoni et al., 2017). The standard therapy for NMIBC is transurethral resection of bladder tumor (TURBT) followed by intravesical therapy (Eifler et al., 2015). For patients with low-risk NMIBC, perioperative single instillation of the chemotherapeutic agent may be considered. Intravesical bacillus Calmette-Guérin (BCG) immunotherapy or intravesical chemotherapy is recommended in patients with intermediate- and high-risk NMIBC (Azevedo et al., 2015). Although BCG is the most effective agent to reduce the risk of recurrence and progression, 50–70% of the tumor recurs within 5 years, and up to 30% of those recurrences progress to muscle-invasive bladder cancer (Dalbagni, 2007; Azevedo et al., 2015). Moreover, the shortage of BCG has continued in the last decade and future shortages are to be expected. Some modifications to the treatment protocol, such as reduction of the instillation dose and number, are alternatives to the current BCG application for NMIBC. However, those modifications may be associated with an increase in the recurrence rate (Fankhauser, et al., 2020). Intravesical chemotherapy after TURBT also decreases the risk of recurrence and progression of NMIBC. Mitomycin C (MMC) is the most common intravesical chemotherapeutic agent. Previous meta-analysis showed that there were no significant differences between BCG and MMC therapy in the progression of the disease, overall survival, and cancer-specific survival (Malmoström et al., 2009). Other chemotherapeutic agents, such as gemcitabine and epirubicin, were compared with BCG therapy in a few clinical trials, and the results suggest inferior efficacy of those agents to BCG (Porena et al., 2010; Sylvester et al., 2010). In order to enhance the efficacy of intravesical chemotherapy, extensive studies have investigated interventional technologies for increasing the dwell time of drugs and cellular uptake of drugs (Douglass and Schoenberg, 2016; Tran et al., 2021). Several clinical trials have shown promising results of electromotive drug administration, drug-releasing implantation, hyperthermia, and photodynamic therapy as drug delivery technologies (Clinicaltrials.gov NCT02307487, NCT03558503, NCT 03945162).

Ultrasound (US)-triggered microbubble cavitation, also known as sonoporation, is a minimally invasive drug delivery technology. Oscillation and collapse of gas-filled microbubbles upon ultrasound exposure transiently increase the cell membrane permeability, which results in the uptake of extracellular molecules by cells (Escoffre and Bouakaz, 2019; Kooiman et al., 2020). Because NMIBC is confined to the surface of the bladder lumen (Tran et al., 2021), US-triggered microbubble cavitation may have the potential for enhancing the efficacy of intravesical chemotherapy in NMIBC patients. Intravesical instillation may enable microbubbles to rise up and attach to the tumor cells that line the surface of NMIBC. Passive regulation of microbubble attachment to cells was achieved by rising bubbles (Sasaki et al., 2012). Stimulation of microbubbles adjacent to tumor cells directly enhances the cellular uptake of chemotherapeutic agents (Lammertink et al., 2016; Sasaki et al., 2017). In addition, US-triggered microbubble cavitation increases tissue penetration of drugs (Nhan et al., 2013) and enhances diffusion of drugs into the tumor cells (Bhutto et al., 2018; Derieppe et al., 2019). Meanwhile, an increase in the permeability of the bladder by US-triggered microbubble cavitation may change the plasma pharmacokinetics of intravesical chemotherapeutic agents. Damages to epithelial cells decreased the transepithelial resistance and significantly increased water and urea permeabilities of the urinary bladder (Lavelle et al., 2002). An advantage of intravesical therapy for NMIBC is that cytotoxic agents are applied to only the bladder lumen and not to normal tissues (Tran et al., 2021). Therefore, it is critical for the development of US-assisted intravesical chemotherapy with minimum invasiveness to restrict the effects of intravesical drugs on the bladder wall.

Herein, we investigated the plasma concentration of an intravesical chemotherapeutic agent in the combination of US-triggered microbubble cavitation. Pirarubicin (4′-O-tetrahydrpyranyl-doxorubicin), an anthracycline derivative, was administered into the urinary bladder of normal dogs. Bladder cancer is the second most common urological cancer in dogs, and bladder cancer in dogs may be a nonexperimental animal model of human bladder cancer (de Brot et al., 2018). Ultrasound-triggered microbubble cavitation was conducted by the intravesical administration of ultrasound contrast agent (Sonazoid) microbubbles and US exposure using a clinical US system. The results of this preliminary safety study suggest that US-triggered microbubble cavitation would be a minimally invasive technology for drug delivery in the urinary bladder.

## Materials and Methods

### Animal

All animal experiments were approved by the Experimental Animals Committee of Hokkaido University (No. 20-0081). Six healthy intact female beagle dogs were enrolled in this safety study. The dogs were owned by the laboratory animal facility of the Graduate School of Veterinary Medicine, Hokkaido University, which is accredited by the Association for Assessment and Accreditation of Laboratory Animal Care International. The median age of the dogs was 3.5 years (range 3–7 years) and body weight ranged between 9.7 and 11.2 kg (median 10.25 kg). The dogs were defined healthy based on history, physical examination, complete blood count, blood biochemistry, urinary analysis, and abdominal sonography. None of the dogs had received any medication and had a history of disease or treatment within the past 6 months.

### Intravesical Chemotherapy

**FIGURE 1:**
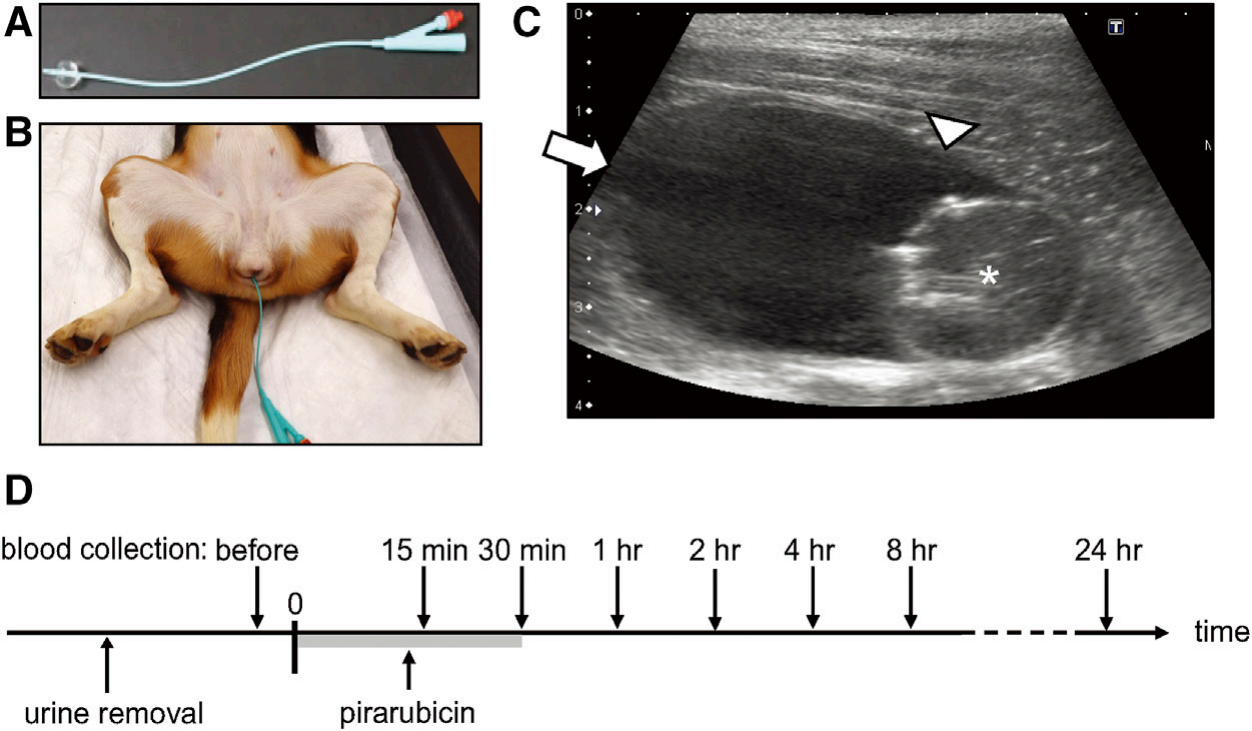
Intravesical chemotherapy. **(A)** Indwelling catheter. **(B)** Catheter was placed in a sedated female beagle dog. **(C)** Representative B-mode image acquired in the sagittal plane. * indicates the catheter balloon. Arrow indicates the apex of the bladder (non-sonicated region), and arrowhead indicates the sonicated region in US-assisted intravesical chemotherapy. **(D)** Timeline of intravesical chemotherapy. Pirarubicin was retained in the urinary bladder for 30 min. Blood was collected before the administration of pirarubicin, and 15, 30 min, 1, 2, 4, 8, and 24 h after the instillation.

The dogs were sedated with an intramuscular injection of 20 µg/kg medetomidine (Domitor, ZENOAQ, Fukushima, Japan). An indwelling catheter (Figure 1A; two-way Foley catheter 8Fr, Create Medic, Kawasaki, Japan) was placed in the sedated dogs, and urine was removed from the bladder (Figure 1B). Ten milligrams of pirarubicin (Nihon-Kayaku, Tokyo, Japan) in 20 ml saline was injected into the bladder *via* the catheter and allowed to dwell for 30 min (Figure 1C). Thereafter, the pirarubicin was removed from the bladder, and the bladder was washed three times using an irrigation of 0.9% sodium chloride (UromaticS, Baxter Limited, Tokyo, Japan).

Plasma pirarubicin concentration during and after a single intravesical instillation was investigated in three dogs. Whole blood was collected before the intravesical instillation and 15, 30 min, 1, 2, 4, 8, and 24 h after the instillation (Figure 1D). Plasma was obtained by centrifuging anticoagulated (EDTA) whole blood. The aliquots of plasma were stored at −80°C until quantification of pirarubicin.

### US-Assisted Intravesical Chemotherapy

**FIGURE 2:**
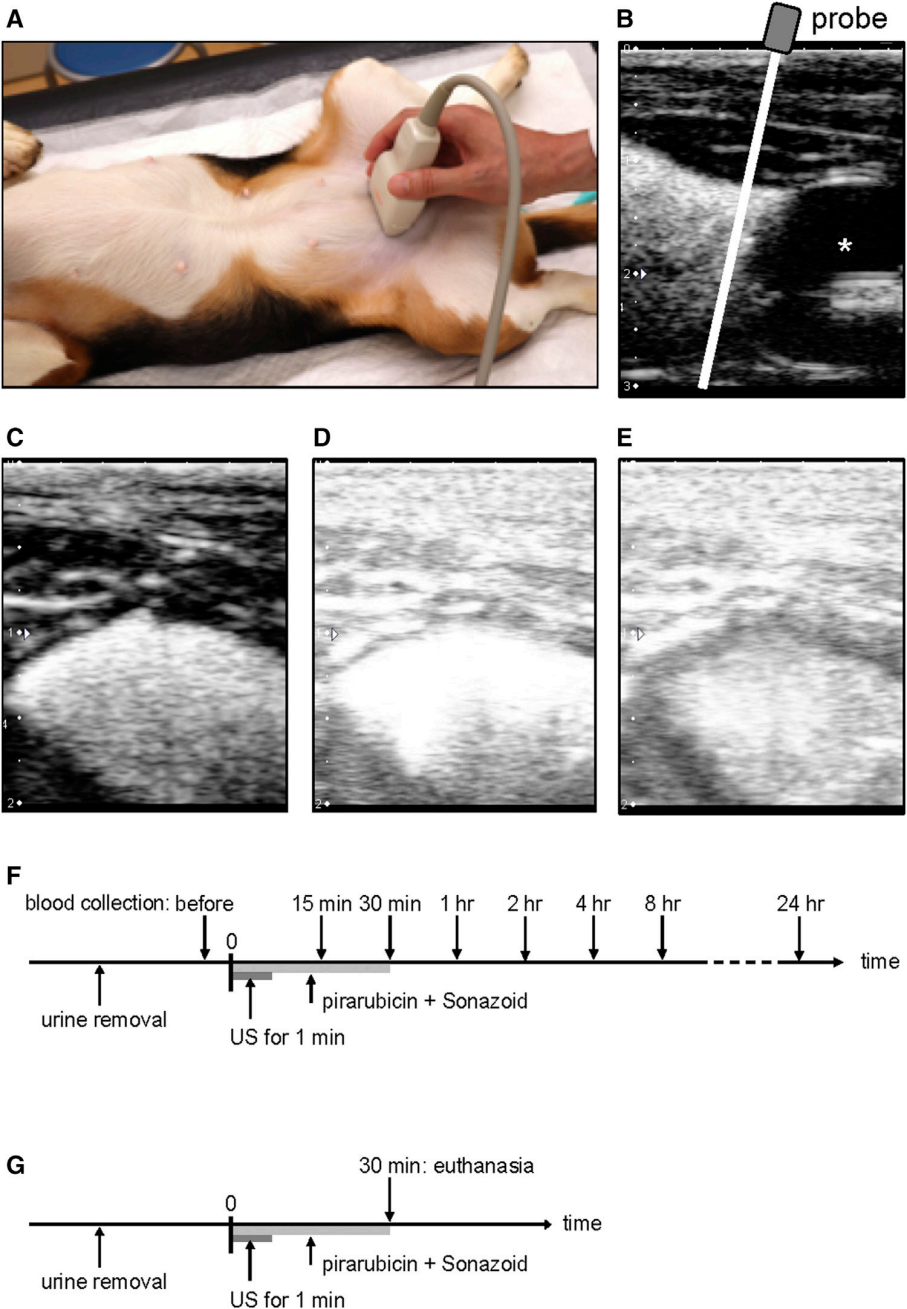
Ultrasound-assisted intravesical chemotherapy. **(A)** Experimental setup. The probe was manually positioned in the transverse plane on a sedated dog. **(B)** Representative image of the bladder in the sagittal plane after microbubble instillation. For the treatment, US was performed on the bladder at the cranial of the catheter balloon using the linear array transducer positioned in the transverse plane. White line indicates a scan plane of the ultrasound during the treatment. **(C)** Representative image before starting the sonication; **(D)** soon after the sonication; **(E)** 1 min after the sonication. **(F)** Timeline of US-assisted intravesical chemotherapy in the plasma pharmacokinetic experiment. Pirarubicin and Sonazoid microbubbles were administered into the bladder, and thereafter ultrasound was performed for 1 min. Pirarubicin was retained in the urinary bladder for 30 min. Blood was collected before the treatment and 15, 30 min, 1, 2, 4, 8, and 24 h after the treatment. **(G)** Timeline of ultrasound-assisted intravesical chemotherapy for histology. Pirarubicin and Sonazoid microbubbles were administered into the bladder, and thereafter ultrasound was performed for 1 min. Pirarubicin was retained in the urinary bladder for 30 min. The dogs were euthanized at the time of 30 min.

The indwelling catheter was placed in the sedated dogs, and urine was removed from the bladder. Pirarubicin (10 mg) in 20 ml saline and 0.1 ml Sonazoid microbubbles (Daiichi-Sankyo, Tokyo, Japan) were injected into the bladder *via* the catheter. The contrast agent comprised a phospholipid shell encapsulating perfluorobutane. Sonazoid was reconstituted in 2 ml of sterilized distilled water according to the manufacturer’s instructions, which contains 1.2 × 10^9^ microbubbles/ml with an average diameter of 3.2 µm (Sontum et al., 1999). Right after the administration, the dogs were positioned in dorsal recumbency and ultrasound was performed on the bladder for 1 min (Figures 2A,B). The mixture of pirarubicin and Sonazoid microbubbles was removed from the bladder 30 min after the US exposure, and the bladder was washed three times using the 0.9% sodium chloride irrigation.

Ultrasound was performed using a linear array probe (PLT-704SBT, Canon Medical Systems, Tochigi, Japan) and a diagnostic US machine (APLIO XG SSA-790, Canon Medical Systems). The probe was operated in a pulse subtraction imaging mode and was manually applied on the abdominal skin of the dogs (Figures 2A,B). The field of view depth was set to 2 cm, and a single focus was placed at a depth of 1 cm from the probe surface (Figures 2C–E). The MI value of 1.33 was indicated on the screen of the diagnostic machine, which is approximately eight-fold higher than that for clinical contrast–enhanced US imaging. Acoustic parameters were as follows: transmitting frequency of 5 MHz, peak-negative pressure of −1.2 MPa, pulse duration of 0.44 μsec, and pulse repetition frequency of 7.7 kHz. The parameters were measured using a membrane hydrophone (MHA500B, NTR Systems, Seattle, WA, United States) in a water tank (Sasaki et al., 2012). The peak negative pressure of the current setup was the strongest in the pulse subtraction imaging mode. During the US exposure, B-mode images were observed on the screen of the diagnostic machine.

Plasma pirarubicin concentration in US-assisted intravesical chemotherapy was evaluated in three dogs. Whole blood was collected before the intravesical instillation and 15, 30 min, 1, 2, 4, 8, and 24 h after the instillation (Figure 2F). Plasma was obtained by centrifuging the anticoagulated whole blood. The aliquots of plasma were stored at −80°C until pirarubicin quantification. In order to measure the pirarubicin concentration in the bladder tissue, another treatment of US-assisted intravesical chemotherapy was conducted 4 weeks after the first treatment (Figure 2G). The dogs were euthanized soon after the treatment, and the bladder was resected. The exposure region of the bladder wall was dissected into two pieces. One piece was stored in −80°C for the quantification of pirarubicin, and the other was fixed in 10% neutral-buffered formalin for the histopathological evaluation. In addition, the apex of the bladder was dissected into two pieces and was analyzed as the nonsonicated region.

**FIGURE 3:**
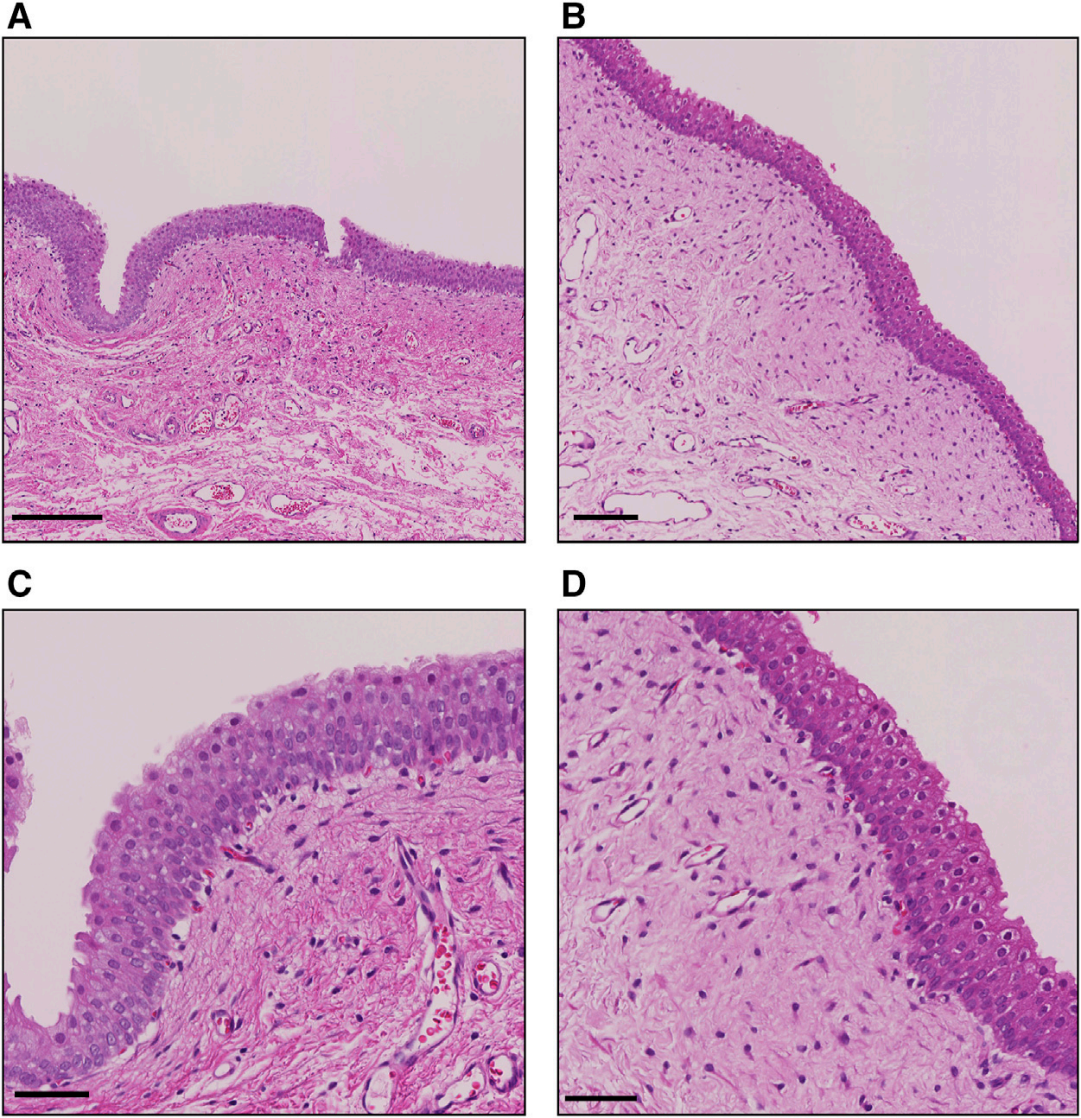
Representative H&E staining images of the bladder after the single treatment. **(A)** Sonicated region at low power field (lpf). bar, 200 µm. **(B)** Nonsonicated region at lpf. bar, 100 µm. **(C)** Sonicated region at high power field (hpf). bar, 50 μm; **(D)** Nonsonicated region at hpf. bar, 50 µm.

Using the other three dogs, US-assisted intravesical chemotherapy was repeated weekly for 9 weeks (Figure 3). The procedures of each treatment were the same with the single US-assisted intravesical chemotherapy. Whole blood was collected before and 30 min after the instillation in each treatment. The dogs were euthanized after the 9th treatment, and the bladder was resected for histological evaluation.

### Pirarubicin Quantification Using LC/MS/MS

Pirarubicin (HPLC grade) was purchased from Sigma-Aldrich (St. Louis, MO, United States). An analytical grade of all reagents and double-distilled water (DDW) was obtained from Kanto-Kagaku (Tokyo, Japan). The quantification of pirarubicin was conducted using an Agilent 6495B Triple Quadrupole LC/MS (Agilent Technologies, Santa Clara, CA, United States). For the measurement of pirarubicin in plasma, 10 µl of plasma was added to 40 µl of 1% formic acid in acetonitrile and mixed vigorously for 5 min. The mixture was centrifuged by a centrifuge (Sorvall ST 8FR, Thermo Fischer Scientific, Waltham, MA, United States) at 10,000 × g for 10 min at room temperature. The supernatant was ultrafiltered using a spin column (MonoSpin® Phospholipid, GL Science, Tokyo, Japan) and the centrifuge at 3,000 × g for 3 min. Thirty microliters of the filtered sample was mixed with 30 µl of 0.1% formic acid in DDW. The samples were injected onto the LC/MS/MS. Separation was performed on an Inert Sustain C18 column (2 μm, 2.0 × 30 mm; GL Science Inc, Tokyo, Japan). The chromatographic gradient program was started at 60% mobile phase A (0.1% formic acid in DDW) with 40% mobile phase B (0.1% formic acid in methanol) at a flow rate of 0.4 ml/min for 3 min. That was followed by a gradient to 100% B for 30 s. The detection was performed with a positive electrospray ionization mode. For the measurement of pirarubicin in tissue, the bladder tissue was homogenized with 1% formic acid in methanol using 5 mg zirconia beads and a homogenizer at 2,500 rpm 3 min × 4 times. The tissue homogenate was centrifuged at 10,000 × g for 10 min. After the supernatant was transferred into another tube, the sediment was re-extracted using the same procedure. The supernatant of the second extraction was mixed with the supernatant of the first extraction. Sixty microliters of the supernatant were mixed with 60 µl of 0.1% formic acid in DDW and applied to the LC/MS/MS quantification.

The standard curves for pirarubicin were obtained using 0.5–100 ng/ml standard solutions. The instrument detection limit was 1 pg, and the method detection limit was 0.5 ng/ml. In addition, the recovery, accuracy, and precision were 87, 4.8, and 5.3%, respectively.

### Histopathology of the Bladder

Histopathology was performed as described previously (Maharani et al., 2018). In brief, the tissues were fixed in 10% neutral-buffered formalin and embedded in paraffin. The paraffin-embedded tissue samples were sectioned to 3-μm films. The sections were stained with hematoxylin and eosin solution (H &E).

### Statistics

Statistical analysis was performed with commercial software (JMP Pro version 14.0, SAS Institute Inc., Cary, NC, United States). The difference in tissue pirarubicin concentration between the sonicated region and nonsonicated region of the same bladder was evaluated using the paired *t*-test. A *p*-value below 0.05 was considered statistically significant.

## Results

Plasma concentration of intravesical pirarubicin was below the lower detection limit in a single US-assisted intravesical chemotherapy and intravesical instillation (Table 1). The peak area of pirarubicin in all plasma samples was lower than that of the lowest pirarubicin concentration in the calibration curve. The tissue concentration of pirarubicin at the sonicated region was higher than that in the non-sonicated region in two of the three dogs, while the concentration did not increase in the other dog (Table 2). The mean tissue concentration of pirarubicin was not significantly different between the sonicated region and nonsonicated region (*p* = 0.09). Figure 3 shows the H&E staining of the bladder wall after the single treatment of US-assisted intravesical chemotherapy. Neither detachment of epithelial cells nor hemorrhage was observed in both the sonicated and non-sonicated regions.

**TABLE 1:** Plasma concentration of pirarubicin (ng/ml) in the single treatment.

		Before	15 min	30 min	1 h	2 h	4 h	8 h	24 h
Intravesical instillation	Dog 1	<0.5	<0.5	<0.5	<0.5	<0.5	<0.5	<0.5	<0.5
	Dog 2	<0.5	<0.5	<0.5	<0.5	<0.5	<0.5	<0.5	<0.5
	Dog 3	<0.5	<0.5	<0.5	<0.5	<0.5	<0.5	<0.5	<0.5
US-assisted intravesical chemotherapy	Dog 1	<0.5	<0.5	<0.5	<0.5	<0.5	<0.5	<0.5	<0.5
	Dog 2	<0.5	<0.5	<0.5	<0.5	<0.5	<0.5	<0.5	<0.5
	Dog 3	<0.5	<0.5	<0.5	<0.5	<0.5	<0.5	<0.5	<0.5

**TABLE 2:** Tissue concentration of pirarubicin after the single US-assisted intravesical chemotherapy.

	Dog 1	Dog 2	Dog 3
Sonicated region	1.65 µg/g-tissue	0.19 µg/g-tissue	2.43 µg/g-tissue
Nonsonicated region	0.21 µg/g-tissue	0.21 µg/g-tissue	1.04 µg/g-tissue

**FIGURE 4:**
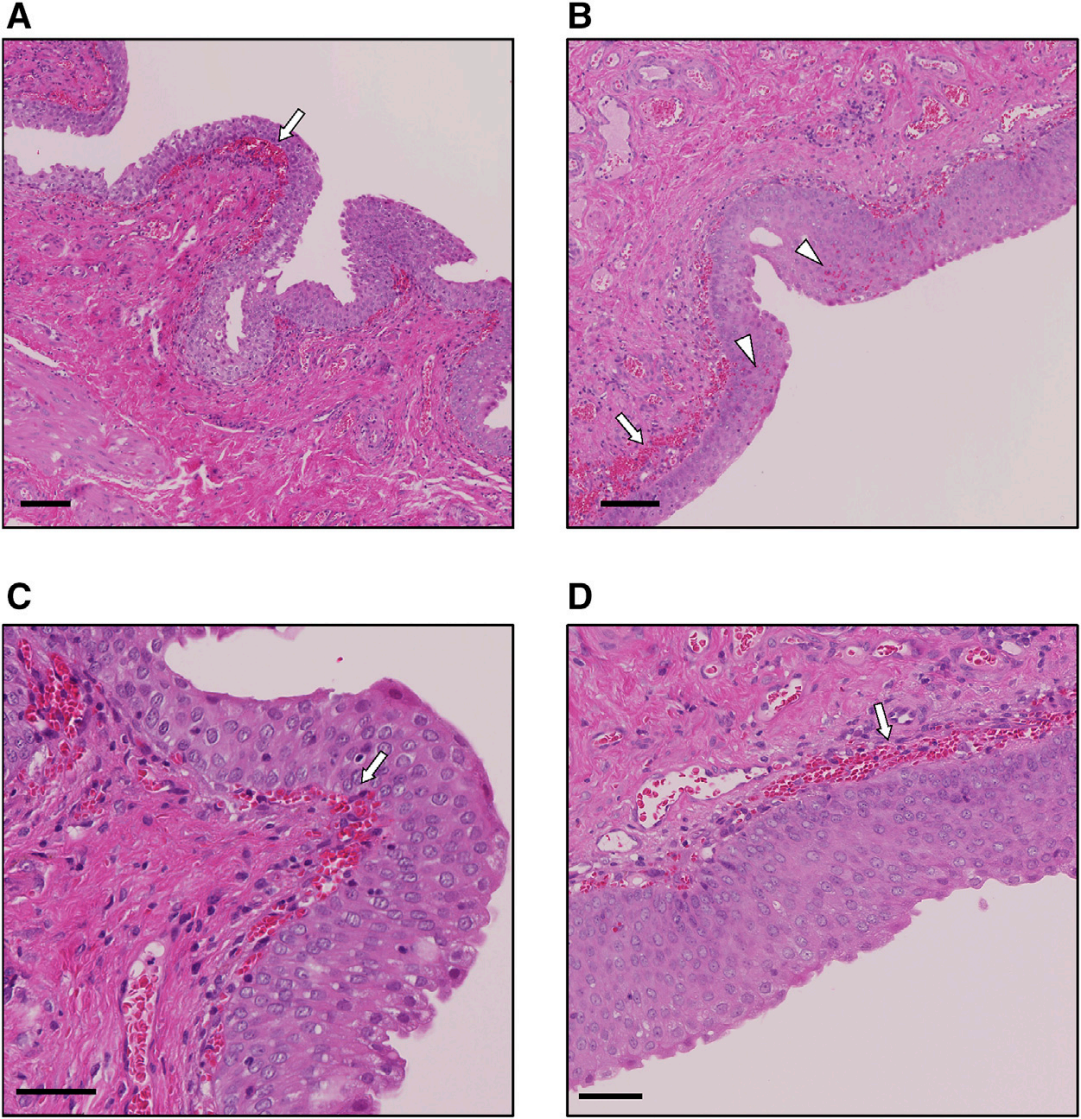
Representative H&E staining images of the bladder after the 9th treatment. **(A)** Sonicated region at low power field (lpf). White arrow indicates congestion. bar, 100 µm. **(B)** Nonsonicated region at lpf. White arrow indicates congestion in the lamina propria, and arrow heads indicate hemorrhages in the urothelium. bar, 100 µm. **(C)** Sonicated region at high power field (hpf). White arrow indicates congestion in the lamina propria. bar, 50 µm. **(D)** Nonsonicated region at hpf. White arrow indicates congestion in the lamina propria. bar, 50 µm.

Pirarubicin was not detected in plasma during the weekly repetition of US-assisted intravesical chemotherapy (Table 3). Figure 4 shows the H&E staining of the bladder after the nine treatments of weekly US-assisted intravesical chemotherapy. The bladder epithelium was thick in both sonicated and non-sonicated regions (Figures 4A,B). In addition, congestion in the lamina propria and mild hemorrhages in the suburothelium were observed in both regions (Figures 4C, D). Indeed, this particular dog showed macrohematuria after the sixth treatment. Macrohematuria in this dog disappeared after additional bladder irrigation at the seventh treatment.

**TABLE 3:** Plasma concentration of pirarubicin (ng/ml) in the weekly US-assisted intravesical chemotherapy.

		1st	2nd	3rd	4th	5th	6th	7th	8th	9th
Before	Dog 4	<0.5	<0.5	<0.5	<0.5	<0.5	<0.5	<0.5	<0.5	<0.5
	Dog 5	<0.5	<0.5	<0.5	<0.5	<0.5	<0.5	<0.5	<0.5	<0.5
	Dog 6	<0.5	<0.5	<0.5	<0.5	<0.5	<0.5	<0.5	<0.5	<0.5
After 30 min	Dog 4	<0.5	<0.5	<0.5	<0.5	<0.5	<0.5	<0.5	<0.5	<0.5
	Dog 5	<0.5	<0.5	<0.5	<0.5	<0.5	<0.5	<0.5	<0.5	<0.5
	Dog 6	<0.5	<0.5	<0.5	<0.5	<0.5	<0.5	<0.5	<0.5	<0.5

## Discussion

The rationale of intravesical therapy is that intravesical drugs have cytotoxic effects on the remaining tumor cells in the bladder lumen and not on normal tissues. Intravesical chemotherapeutic agents, such as mitomycin C and gemcitabine, are absorbed into the blood (Dalton et al., 1991; Campodonico et al., 2007). Meanwhile, the systemic absorption of pirarubicin is not detected in intravesical instillation after TURBT for patients with NMIBC (Arakawa et al., 2011). The rapid uptake of pirarubicin by tumor cells may contribute to the less systemic absorption (Kunimoto et al., 1983). The increase in systemic absorption of pirarubicin by US-triggered microbubble cavitation was not prominent in this preliminary study. Previous studies showed that the penetration distance of nanoscale agents by US-triggered microbubble cavitation in *in vivo* models was up to 80 µm depending on the acoustic intensity and size of agents (Theek et al., 2016; Yemane et al., 2019; Olsman et al., 2020). Therefore, it may be possible that US-triggered microbubble cavitation increases the extravasation of pirarubicin into the bladder wall, and thereafter pirarubicin rapidly diffuses into epithelial cells. However, tissue distribution of pirarubicin was not investigated in this study. Eight additional weekly instillations of pirarubicin reduced the recurrence rate in NMIBC patients with intermediate risk (Naya et al., 2018). Ultrasound-assisted intravesical chemotherapy was repeated nine times weekly, and pirarubicin was not detected in plasma at any time points. In addition to the single treatment, weekly repetition of US-assisted chemotherapy may not cause systemic side effects. Local side effects of intravesical pirarubicin are frequent urination, pain on urination, and macrohematuria (Okamura et al., 2002; Naya et al., 2018; Fujita et al., 2020). The incidence of local adverse effects in patients with pirarubicin maintenance therapy (e.g., eight additional weekly instillations) was higher than in patients with single intravesical instillation of pirarubicin (Naya et al., 2018). Although multiple intravesical chemotherapy without sonication was not conducted in this study because of the ethics in animal experiments, histopathology of the bladder wall showed hemorrhage and congestion at the nonsonicated region in the multiple treatments. Therefore, it is likely that macrohematuria was an adverse effect of the repeated intravesical administration of pirarubicin.

A few clinical studies showed the feasibility of ultrasound-mediated drug delivery using clinical diagnostic scanners for cancer treatment (Dimcevski et al., 2016; Wang et al., 2018; Eisnebrey et al., 2021). However, limited information was available for the increase in the tissue concentration of the delivered chemotherapeutic agents. The tissue concentration of pirarubicin in NMIBC patients after the single intravesical instillation varies in a wide range (Arakawa et al., 2011). Dilution by urine and change in urine pH are possible mechanisms of the wide range of drug concentrations in the bladder tumor tissue (Gontero et al., 2010). Further clinical studies should be needed to quantify the amount of delivered pirarubicin by US-assisted intravesical chemotherapy in NMIBC patients and dogs with bladder cancer.

A few studies investigated the potential application of US-triggered microbubble cavitation to intravesical treatment (Horsley et al., 2019; Ruan et al., 2021). Ruan et al. evaluated the efficiency of gemcitabine delivery by the combination of US and drug-loading microbubbles in a mouse orthotopic bladder cancer model of muscle-invasive bladder cancer (Ruan et al., 2021). Horsley et al. proposed antibiotic delivery using US-triggered microbubble cavitation for urinary tract infection (Horsley et al., 2019). In addition to those applications, our preliminary results suggest that US-assisted intravesical chemotherapy for NMIBC may have a promising application of US-mediated intravesical therapy. Treatment protocol, including intravesical doses and US parameters, should be optimized for facilitating the translation of US-triggered microbubble cavitation from the animal models to the clinics. The pirarubicin dose and dwell time in the current protocol would be relevant to clinical treatment. A human equivalent dose of 10 mg pirarubicin in dogs is 0.54 mg/kg according to a dose translation formula (Reagan-Shaw et al., 2007). Pirarubicin is administered at the dose of 30 mg (1 mg/ml) and retained for 1 h in patients with NMIBC. We used a clinical US system (Cannon Aplio-XG) and clinical microbubbles (Sonazoid). Ultrasound parameters in the current setup were previously investigated for drug delivery in *in vitro* and *in vivo* studies (Sasaki et al., 2012; Sasaki et al., 2014). Moreover, de Maar et al. demonstrated that delivery efficiency using a clinical US system would be comparable to that of a custom-built US system with optimized parameters (de Maar et al., 2021). Therefore, the treatment protocol of this preliminary study may be applicable to an efficacy study. Future clinical studies should assess the efficacy of US-assisted intravesical chemotherapy in dogs with bladder cancer and in patients with NMIBC.

## Data Availability

The raw data supporting the conclusions of this article will be made available by the authors, without undue reservation.
